# Myeloid-Derived Suppressor Cells in Multiple Myeloma: Pre-Clinical Research and Translational Opportunities

**DOI:** 10.3389/fonc.2014.00348

**Published:** 2014-12-08

**Authors:** Cirino Botta, Annamaria Gullà, Pierpaolo Correale, Pierosandro Tagliaferri, Pierfrancesco Tassone

**Affiliations:** ^1^Department of Experimental and Clinical Medicine, “Magna Graecia” University and Medical Oncology Unit, T. Campanella Cancer Center, “Salvatore Venuta” University Campus, Catanzaro, Italy; ^2^Unit of Radiotherapy, Siena University Hospital, Siena, Italy; ^3^Sbarro Institute for Cancer Research and Molecular Medicine, Center for Biotechnology, College of Science and Technology, Temple University, Philadelphia, PA, USA

**Keywords:** MDSC, myeloma, immunosuppression, cancer, pre-clinical models

## Abstract

Immunosuppressive cells have been reported to play an important role in tumor-progression mainly because of their capability to promote immune-escape, angiogenesis, and metastasis. Among them, myeloid-derived suppressor cells (MDSCs) have been recently identified as immature myeloid cells, induced by tumor-associated inflammation, able to impair both innate and adaptive immunity. While murine MDSCs are usually identified by the expression of CD11b and Gr1, human MDSCs represent a more heterogeneous population characterized by the expression of CD33 and CD11b, low or no HLA-DR, and variable CD14 and CD15. In particular, the last two may alternatively identify monocyte-like or granulocyte-like MDSC subsets with different immunosuppressive properties. Recently, a substantial increase of MDSCs has been found in peripheral blood and bone marrow (BM) of multiple myeloma (MM) patients with a role in disease progression and/or drug resistance. Pre-clinical models recapitulating the complexity of the MM-related BM microenvironment (BMM) are major tools for the study of the interactions between MM cells and cells of the BMM (including MDSCs) and for the development of new agents targeting MM-associated immune-suppressive cells. This review will focus on current strategies for human MDSCs generation and investigation of their immunosuppressive function *in vitro* and *in vivo*, taking into account the relevant relationship occurring within the MM–BMM. We will then provide trends in MDSC-associated research and suggest potential application for the treatment of MM.

## Introduction

The immune system has the potential to selectively kill tumor cells avoiding normal tissue and to generate long-lasting memory, which prevents cancer onset or recurrence. However, even if some patients achieve long-lasting complete remissions, the clinical efficacy of immunotherapy is still limited. One possible explanation should be identified in the protective *milieu* provided by tumor-associated inflammation and tumor-infiltrating myeloid and lymphoid cells (1, 2). The critical role of inflammation in cancer development and progression has been recognized over 150 years ago by Rudolf Virchow; however, only recently, chronic inflammation has been found to induce immunosuppression and has been associated with the development of cancer and other diseases (3–5). In the inflammatory response, myeloid effectors are the first cells attracted and recruited in the site of injury (6). Indeed, in cancer, these cells are represented by neutrophils, macrophages, dendritic cells (DCs), and the highly immunosuppressive myeloid-derived suppressor cells (MDSCs) (7). MDSCs are a heterogeneous population of immature myeloid cells generated in the bone marrow (BM) of healthy subjects that normally differentiate in mature myeloid cells without inducing immunosuppression (7). However, in pathologic conditions such as cancer, trauma, or other diseases characterized by chronic inflammation, these cells undergo abnormal expansion, are blocked in differentiation and accumulate in different sites including BM, spleen, liver, and tumor site (8, 9), sustaining (or even worsening) preexisting tumor-driven inflammation and inducing tumor-progression, neovascularization, and immune-escape (7, 10–12).

In the last decade, several studies have focused on the role of MDSCs in the regulation of immune system in solid tumors while little is reported on the role of MDSCs in hematologic malignancies, including multiple myeloma (MM) (13). MM is an incurable disease characterized by accumulation of malignant plasma cells within the BM. The interplay between MM cells and cells of the BM microenvironment (BMM) is tough to be the cause, of two widely recognized hallmarks of MM: bone disease and general immunosuppression (14–16). Recently, it has been disclosed a major role for MDSCs in MM pathobiology. Indeed, different authors demonstrated an increase of MDSCs in both peripheral blood and BM of MM patients (9, 17, 18). Moreover, it was reported that in addition to their immunosuppressive activity, MDSCs have the potential to differentiate in functional osteoclasts thus contributing to the formation of osteolytic lesions (19, 20).

This review provides an overview on current pre-clinical approaches used to study human MDSCs *in vitro* and *in vivo*, with a particular focus on MM-associated MDSCs. We will also summarize the mechanism and the molecular pathways involved in MDSC-dependent immune-dysfunction and potential translational applications of MDCSs for the therapy of MM.

## Immunophenotype of MDSCs

Myeloid-derived suppressor cells were firstly identified in tumor-bearing mice on the basis of their suppressive function. Indeed, these cells lacked any surface marker specifically expressed by myeloid mature cells such as monocytes, macrophages, or DCs (21), even if morphologically they resembled granulocytes or monocytes. Further works in mice, aimed to characterize their immunophenotype, identified MDSCs as cells positive to granulocyte receptor (Gr1) and CD11b (22). The mAb used to identify Gr1, however, is able to bind the same epitope of two different molecules belonging to the lymphocyte superfamily (Ly)-6, Ly-6C, and Ly-6G, mainly expressed on monocytes and neutrophils, respectively. This led to the identification of two sub-groups of murine MDSCs, reflecting differences in term of both morphology and immunosuppressive function: CD11b^+^ Ly-6C^+^ Ly-6G^−^ monocytic (Mo)-MDSCs, with a monocyte-like morphology, mainly expressing the inducible form of nitric oxide synthase (iNOS); and CD11b^+^ Ly-6C^−^ Ly-6G^+^ granulocytic (G)-MDSCs [or polymorphonuclear (PMN)-MDSCs] with a granulocyte-like morphology expressing high levels of arginase 1 (ARG1) (23, 24).

Unlike murine MDSCs, the human MDSCs are less defined, lacking a Gr1 homologous. Commonly, MDSCs are defined as CD11b^+^ CD33^+^ HLA-DR^low/−^ cells not expressing markers of mature myeloid or lymphoid cells. As in mice, two main subsets of MDSCs could be identified: CD15^+^ CD14^−^ CD11b^+^ CD33^+^ HLA-DR^low/−^ G-MDSCs and CD15^−^ CD14^+^ CD11b^+^ CD33^+^ HLA-DR^low/−^ Mo-MDSCs (7, 25, 26). Furthermore, human MDSCs are extremely heterogeneous and recently different tumors were reported to generate MDSCs identified by different phenotypes (27). New markers are currently under investigation in order to better clarify their phenotype, subsets, and function. Among them, the expression of IL-4Ra and the VEGF receptors 1 and 2, on Mo-MDSCs in particular, have been associated with immunosuppressive and angiogenetic potential, respectively (7, 22). As regard to morphology, human MDSCs resemble granulocytes or monocytes at different maturation stages (28) and in cancer patients their number have been reported to directly correlate with neutrophil count (29). Taking into account the prognostic and predictive role played by neutrophil count and chronic inflammation in different malignancies (30–33), the key role of these cells in cancer patients is an emerging issue.

In MM, few reports (9, 17, 18) identified MDSCs as CD11b^+^ CD33^+^ HLA-DR^low/−^ cells in both peripheral blood and BM. G-MDSC resulted to be the most up-regulated and immunosuppressive subpopulation, while contrasting results were observed regarding the Mo-MDSCs. This should be due to differences in phenotypic profile, which identifies Mo-MDSCs. Indeed, while most of authors recognized G-MDSCs as CD15^+^ CD14^−^ CD11b^+^ CD33^+^ HLA-DR^low/−^ cells, Mo-MDSCs have been alternatively identified as CD14^+^ CD11b^+^ HLA-DR^low/−^ (9, 18) or CD14^+^ CD11b^+^ CD33^+^ HLA-DR^low/−^ (17) or CD15^−^ CD14^−^ CD11b^+^ CD33^+^ cells (9). Of note, in a previous report (34), Mo-MDSCs identified as CD14^+^ HLA-DR^low/−^ cells were found to be significantly higher in patients with MM as compared to healthy subjects.

On these bases, due to the lack of a unique surface markers signature for the identification of MDSCs, functional suppressive assays remain essential.

## MDSCs Function and Role in Tumor-Progression

**Figure 1:**
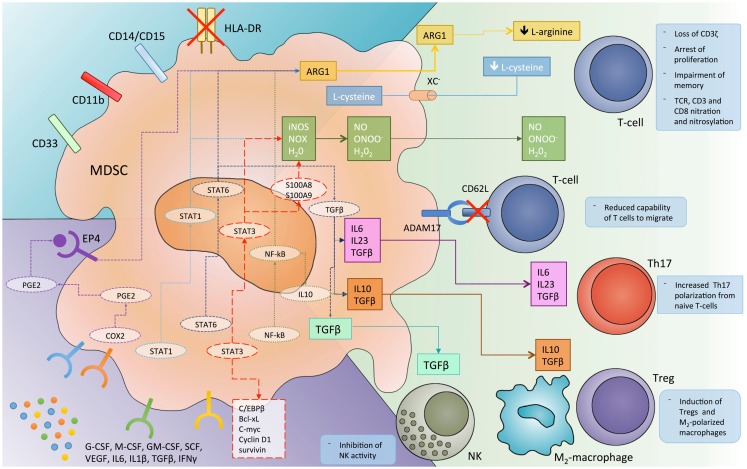
**The different mechanisms by which myeloid-derived suppressor cells (MDSC) inhibit immune system response and the molecular pathways involved in this immunosuppressive function**. ADAM17, disintegrin and metalloproteinase domain-containing protein 17; ARG1, arginase 1; C/EBPβ, CCAAT/enhancer-binding protein-β; COX, cyclooxygenase; EP4, prostaglandin receptor E4; iNOS, inducible nitric oxide synthase; NK, natural killer cells; NO, nitric oxide; NOX, NADPH oxidase; PGE2, prostaglandin E2; ROS, reactive oxygen species; STAT, signal transducer and activator of transcription; TCR, T cell receptor; TGFβ, transforming growth factor-β; T-regs, regulatory T cells; VEGF, vascular endothelial growth factor; Xc^−^, cystine–glutamate transporter.

Myeloid-derived suppressor cells inhibit the anti-tumor immune response by multiple mechanisms, probably mostly triggered by direct cell-to-cell contact and involving cell surface receptors and short-lived mediators (Figure 1). Among others, the metabolism of L-arginine was the first identified mechanism for MDSCs immunosuppression (35, 36). Specifically, L-arginine serves as a substrate for iNOs (generating NO and citrulline) and ARG1 (producing urea and ornithine). The up-regulation of both enzymes in MDSCs lead to a shortage of the non-essential amino acid in tumor microenvironment, and consequently to the impairment of T cell function. Indeed, T lymphocytes depend on arginine for proliferation, CD3ζ expression, and development of memory (37, 38). Furthermore, the increased NO production leads to suppression of T cell function through the inhibition of IL-2 downstream pathway (39, 40). An additional and related finding is that, MDSCs could also mediate the depletion of cystine and cysteine from tumor microenvironment, thus further limiting antigen-driven T cell activation (41).

A further hallmark of MDSC-dependent immunosuppression relies on the production of reactive oxygen species (ROS). Indeed, different studies report MDSCs from both tumor-bearing mice and cancer patients to produce a huge amount of ROS (23, 42, 43) that, in turn, reduce CD3ζ expression and antigen specific T cell proliferation (44). ROS production by MDSC is sustained by the inflammatory tumor microenvironment, enriched, among others, in IL-10, IL-6, and TGF-beta, as well as by the cell-to-cell contact with lymphocytes (37, 44).

The contemporary presence of NO and superoxide in tumor microenvironment may lead to the production of peroxynitrite, which in turn induces the nitration of different amino acids such as tyrosine, tryptophan, cysteine, and methionine (45). Nitration of T cell receptor (TCR) and CD8 molecules results in a physical modification of the receptor that alters the binding to MHC, thus impairing the capability of T cells to respond to antigen specific stimuli (46).

Recently, some authors (47) reported a new mechanism by which MDSCs inhibit the immune response against cancer. They found that MDSCs are able to down-regulate L-selectine (CD62L) levels on naïve T cells through their membrane expression of ADAM17. This event, in turn, decreases the capability of T cells to migrate to the tumor site where they would be activated.

A further mechanism of immune regulation by MDSCs relies on their capability to induce regulatory T cells (T-regs). Indeed, different studies report that MDSCs promote the clonal expansion of antigen specific natural T-regs and induce the conversion of naïve T helper cells into inducible T-regs, through a mechanism dependent on CD40-CD40L interaction; on the secretion of cytokines such as IFN-gamma, IL-10, and TGF-beta; and on the overexpression of ARG1 (37, 48–50).

More recently, MDSCs have been reported to promote Th17 differentiation and IL-17A production (51). Th17 development was shown to be dependent on IL-1b/IL-6/IL-23 and NO production by MDSCs in both tumor-bearing mice and cancer patients (52–54). To better understand the paradoxical induction of “pro-inflammatory” Th17 by “immunosuppressive” MDSCs, it should be noted that Th17 cells play a role in both tumor-progression and induction of immunosuppression. Indeed, these cells promote chronic inflammation, DNA damage, and tumor-associated angiogenesis, and on the other hand promote the local recruitment of other inflammatory cells (including MDSCs) and inhibit immune response (51, 55, 56).

A further immunosuppressive mechanism relies on the cross-talk between MDSCs and tumor-associated macrophages (TAMs) (57, 58). This strict interplay leads to a microenvironment enrichment in immune-regulatory cytokines such as IL-10, IL-6, IL-1b, VEGF, and to an immunosuppressive M2 polarization of macrophages. These cells also promote tumor-progression through different non-immune mechanism and in MM, in particular, are reported to induce cancer cell proliferation, drug resistance, and angiogenesis (59, 60).

Finally, emerging evidence suggests that MDSCs may promote immune-escape also by suppressing NK activity, overexpressing programmed death ligands (PD-L1 and PD-L2) and releasing IL-10 and indoleamine 2,3-dioxygenase (IDO) in the tumor microenvironment (37, 61, 62).

## Molecular Regulators of MDSCs Development and Function

The tumor-associated microenvironment produces several factors involved in myelopoiesis and impairs the myeloid differentiation. Among them, granulocyte-macrophage colony-stimulating factor (GM-CSF), G-CSF, M-CSF, stem cell factor, VEGF, and IL-3 are the most recognized molecules involved in MDSC generation (27, 63). Furthermore, a huge amount of cytokines and chemokines released by tumor cells or tumor-surrounding cells including IL-1β, IL-4, IL-6, IL-10, IFN-gamma, TGF-beta, CCL2, CCL5, S100A8, and S100A9, are reported to reprogram immature myeloid cells to became immunosuppressive MDSCs and to attract them in the tumor microenvironment (7, 64, 65). All these soluble mediators regulate MDSC function through the activation of different downstream signaling pathways, such as Jak/Stat, NF-κB, cyclooxygenase 2 (COX-2), and PGE2 (27, 66). Furthermore, recent evidence supports the hypothesis that a major role in this regulatory network is played by non-coding RNAs including micro-RNAs (miRNAs) and long non-coding RNAs (lncRNAs) (67–69).

### STAT family

The signal transducer and activator of transcription (Stat) 3 plays, among other Stat family members, a major role in modulating MDSC function. In myeloid cells, Stat3 promotes the expression of different anti-apoptotic and pro-proliferative factors, such as Bcl-xL, c-myc, cyclin D1 and survivin, and prevent myeloid cells differentiation and maturation (66, 70). Stat3 induces the up-regulation of the calcium-binding pro-inflammatory proteins S100A9 and S100A8. Some authors reported that Stat3-dependent S100A9 up-regulation enhances MDSC generation *in vitro* and that the immune system of mice lacking S100A9 have a greater ability to reject the tumor implant (71). The mechanism is still not fully understood, however, it is thought that the heterodimer S100A8/S100A9 is involved in ROS generation through NAPDH oxidase (Nox2) complex and that ROS, in turn, impairs myeloid cells differentiation (71). Moreover, STAT3 up-regulates two components of the Nox2 complex, p47^phox^, and gp91^phox^, thus directly contributing to the increase in ROS production by MDSCs (72). Stat3 is also reported to interact with C/EBPβ, a transcription factor involved in myelopoiesis and control of differentiation and proliferation of myeloid progenitors. The latter could be explained, at least in part, by the capability of activated Stat3 to induce c-myc expression due to the increased binding of C/EBPβ to Myc promoter (65, 73).

Among the other members of the Stat family, Stat1 and Stat6 play a key role in MDSC activation and immunosuppressive function. Stat1 is activated by both IFN-γ and IL-1β and is involved in the expression of ARG1 and iNOS. Indeed, MDSCs from mice knock-down for STAT1 are unable to inhibit T lymphocyte activation due to a lack in up-regulation of iNOS or ARG1 (74).

IL-4 or IL-13 binding to the CD124 leads to the activation of Stat6, which, in turn, induces the expression of arginase and the production of TGFβ by MDSCs, thus contributing to the instauration of an immune-permissive microenvironment (75–77).

### NF-κB

The NF-κB activity has been reported to be critical for the immunosuppressive capability of MDSCs. Toll-like receptors (TLR) via myeloid differentiation primary response gene (MyD) 88 and IL-1β are fundamental activators of NF-κB signaling and lead to the production and secretion of Th2 cytokines, IL-10, and ARG1 (66). Recently, it has been demonstrated (78) a Stat3-dependent activation of the non-canonical NF-κB pathway that, in turn, induces the transcription of IDO in MDSCs, thus elucidating a new mechanism for T cell immunity inhibition.

### PGE2 and COX-2

Prostaglandin E2 is an eicosanoid that act as both pro-inflammatory and immunosuppressive molecule. Its synthesis is COX-2 dependent and its signaling relies on the receptor E-prostanoid (EP) 4, which once activated induce ARG1 up-regulation in MDSCs (79–81). EP2-knockout mice inoculated with 4T1 mammary carcinoma presented a reduced number of infiltrating MDSCs, suggesting an important role for PGE2 in MDSC induction (66). Furthermore, others provided evidence (82) that the COX-2 inhibition, which is induced with dietary celecoxib in a mesothelioma murine model, prevents the expansion of MDSCs. Together these results highlight the crucial role played by the COX-2/PGE2 signaling in MDSCs function and differentiation.

### Micro-RNAs

Micro-RNAs are small non-coding RNAs able to regulate gene expression at the post-transcriptional level (83). Due to their critical role in tumor biology (84–86) and cancer–microenvironment interaction (87–90), these molecules have gained particular attention even as possible immune modulators (91, 92). Indeed, different studies identified several miRNAs involved in the complex network that regulate MDSCs development and function (67, 68).

MiR-223 was the first miRNAs associated to MDSCs (93). It has been discovered that increased PGE2 in the tumor microenvironment leads to miR-223 down-regulation in MDSCs and subsequent up-regulation of its target myeloid enhancer factor 2 (Mef2c), which promote MDSC survival and accumulation in tumor site (93). MiR-223 is involved also in myeloid cell differentiation by targeting nuclear factor I (NFI)-A and inducing differentiation of immature myeloid cells into mature granulocytes (94).

MiR-494 has been shown to be up-regulated in MDSCs by tumor-derived factors (especially from TGF-β). This miRNA revealed to sustain MDSC survival through down-regulation of PTEN and to promote tumor invasion and metastasis by regulating metalloproteinase (MMP) expression (95).

Contrasting observations were reported for miR-17-5p and miR-20a by two different studies. Specifically, some authors reported (96) that both miRNAs target AML1, leading to a down-regulation of M-CSF receptor and, consequently, to an impairment of monocyte differentiation. On the contrary, others demonstrated (97) that the tumor microenvironment led to both miRNAs down-regulation and that, according to their capability to target Stat3, their reduction leads to the up-regulation of Stat3 itself and the activation of the Stat3-dependent immunosuppressive cascade.

Using miRNA microarray, miR-21 and miR-155 have been identified as the two most up-regulated miRNAs during the induction of MDSC differentiation from BM cells (98). This up-regulation was further confirmed in tumor-bearing mice with a mechanism dependent on the presence of TGFβ in tumor microenvironment. By targeting SHIP-1 and PTEN, respectively, these miRNAs lead to Stat3 activation and MDSC proliferation and activation. Conversely, melanoma and Lewis lung carcinoma grew faster in miR-155 knockout mice and these tumors showed an increased MDSC infiltration (99). This event appears dependent on the up-regulation of HIF-1α (a direct target of miR-155) in miR-155^−/−^ mice that leads to the enhanced expression of different chemokines and cytokines promoting MDSC recruitment, immunosuppression, and neovascularization. These apparent conflicting results may be dependent on the great heterogeneity of MDSCs and on the capability of different tumors to induce predominantly one rather than another MDSC subset. Further studies will better elucidate the role of miRNAs in MDSC-mediated cancer-associated immunosuppression.

## *In vitro* and *In vivo* Approaches for Human MDSCs Study in Multiple Myeloma

It is becoming clear that the long-term success of cancer immunotherapy depends on the reprograming of the tumor-associated immune-permissive microenvironment. The great immunosuppressive potential of MDSC coupled with their capability to accumulate in tumor tissue, makes them attractive targets for the development of specific anticancer therapy. However, this relevant challenge requires the development of a stable model for the study of human MDSC response to different experimental conditions. *In vitro*, generation of murine MDSCs is a relatively easy process that uses embryonic, splenic, or BM myeloid progenitor cultured in a medium enriched with a combination of growth factor such as GM-CSF + G-CSF, GM-CSF + IL-6, or IL-13 (100, 101). However, this model is hampered by the interspecies differences in both surface marker and biology of immune cells (102); additionally, the possibilities to reproduce this model with human cells are low due to the poor availability of myeloid progenitors from healthy donors.

Recently, to overcome these issues, some authors developed a method to generate MDSCs from healthy donors’ peripheral blood mononuclear cells (PBMCs) (103, 104). As a first step of development of their model, authors evaluated the capability of 100 tumor cell lines to induce MDSC generation from healthy donor PBMCs during a 7-day co-culture. MDSCs so generated were phenotypically characterized and the CD33^+^ CD11^+^ HLA-DR^−/low^ population was sorted and assayed for the capability to suppress autologous T cell proliferation in response to stimuli (104). A cytokine study was simultaneously performed to identify the main factors responsible for MDSCs’ generation (103). The authors observed that a 7-day culture with the combination GM-CSF + IL-6 was sufficient to generate a potent immunosuppressive CD33^+^ MDSC population. Of note, MDSCs were also generated, even if to a lesser extent, by combining GM-CSF with cytokines such as IL-1β, VEGF, TNFα, and PGE2, providing thus evidence that the presence of an inflammatory microenvironment is mandatory for MDSC generation. Furthermore, cytokine-induced MDSCs, as their tumor-induced counterpart, cause immunosuppression through the up-regulation of ARG1, iNOS, VEGF, and TGFβ (103).

In subsequent years, this model has been used to demonstrate the capability of different MM cell lines to induce, *in vitro*, the generation of functional MDSCs from healthy donor PBMCs and from BM aspirates (18).

Unlike *in vitro* studies, all models currently available for *in vivo* study of MDSCs are murine models. Furthermore, only two different mouse models have been used to investigate the role of MDSCs in MM. One of them is the syngeneic 5TMM mouse model inoculated with either the 5T2MM or the 5T33MM cell lines (105). These cells home in the BM and recapitulate very closely the human disease (including BM–MM cells interactions and osteolytic lesions) (105). This was the first demonstration of MDSC presence and activity in MM.

The second model was obtained inoculating mice with syngeneic murine BCM, DP 42, and ATLN MM cell lines (9). Cells in this model home to BM, and allow investigation of MDSCs in the BM *milieu*. By using these models, the authors discovered that MDSC infiltration of BM occurs early after tumor inoculation while, in the subsequent weeks, the percentage of infiltrating MDSC slowly decreases due to the increase of malignant PCs. Furthermore, by using S100A9 knockout transgenic mice, which have an impaired MDSC response to cancer, it has been observed a significant delay in tumor growth, which was reverted by adoptive administration of MDSCs, thus evidencing the key role played by MDSCs in tumor escape from immune system. However, the lack of models that recapitulate the complex human MM *milieu* limits the study of human MDSC interaction with MM cells and other component of BM *milieu*, thus limiting, in a translational view, the study of agents that may target specifically human MM-associated BM cells (106).

The SCID-hu model has been realized in an attempt to overcome these limitations (107–110). In this model, a human fetal bone chip is implanted in SCID mice and subsequently, primary patient malignant cells or BMSC-dependent human plasma cell line INA-6 are injected directly into the human bone implant. This model demonstrated to be suitable and reliable system to evaluate the anti-tumor activity of different drugs including different anti-inflammatory agents (108–116). This model appears to be a good candidate for the study of MDSCs, even if some immunological pitfalls should be taken into account: the allogeneic nature of BM cells respect to both primary cells or MM cell lines and the bone chips heterogeneity due to different gestational age at which they are collected (106). Both these caveats have been overcome by the use of the SCID-*synth-hu* model (117). This model is based on the implantation of a tridimensional bone-like polymeric scaffold into a SCID mouse and on the subsequent injection of the whole unselected cell population from BM aspirates into the implanted scaffold. This model has been successful used for pre-clinical evaluation of a variety of investigational agents (83–85, 106, 117). The recapitulation of an autologous BMM potentially offers the best model to investigate the MM-associated immunosuppressive niche and the strict interplay between MM cells and immature myeloid progenitors, including MDSCs, thus representing a unique tool for the development and evaluation of immune-modulating agents in MM.

## Translational Opportunities in MM

Recently, MDSCs have been associated with immune-dysfunction in MM patients. Few reports (9, 17, 18) demonstrated the great complexity of the microenvironment in which pro-inflammatory factors co-exist with immune-suppressive mediators, in a finely tuned balance that influences patients’ outcome (118–120). Additionally, different pro-inflammatory cytokines including IL-1β, IL-6, IL-17, TNFα, and IL-23 as well as anti-inflammatory cytokines such as IL-10, TGFβ, or VEGF have been reported to be up-regulated in MM patients in both peripheral blood and BM (1, 121–123). This peculiar BMM, on one hand potently impairs the capability of resident myeloid progenitors to differentiate into DCs, macrophages, or granulocytes, and on the other reprograms mature myeloid cells to assume an immune-permissive phenotype (tolerogenic DCs, M2, N2) (7). Obviously, these events lead to a vicious circle in which MM cells drive inflammation and immunosuppression while both sustain and promote tumor growth (2, 124). To interrupt this sequence of events, different drugs are under investigation or already available in MM and other malignancies that may potentially interfere with MDSC activity and different steps (Table 1).

**Table 1:** **Pre-clinical and clinical agents targeting MDSCs**.

Differentiation and expansion	Intracellular modulators	Function	Depletion
ATRA	IL-6R blockers	ARG1 and iNOS inhibitors	Chemotherapeutic agents
HDAC inhibitors	JAK inhibitors	PDE-5 inhibitors (tadalafil, sildenafil)	Capecitabine
Blocking cytokines	STAT3 inhibitors	COX-2 inhibitors	Gemcitabine
IL-1β (anakinra)	miRNAs	Celecoxib	Doxorubicin
IL-6		ROS inhibitors	IL-4Rα aptamer
VEGF (bevacizumab)		Nitroaspirin	Peptibodies
Blocking hematopoietic growth factor		N-Acetyl cysteine	
G-CSF		Bisphosphonates	
M-CSF		Zoledronate	
GM-CSF			

Histone-deacetylase inhibitors are a novel class of drugs involved in the epigenetic modulation of gene expression, which revealed to have great anti-tumor activity in MM (125, 126). Additionally, these agents revealed different anti-inflammatory properties related to their capability to impair, among others, the IL-6/Jak/Stat and the NF-κB signaling (127). Some authors recently reported that the histone-deacetylase inhibitor (HDAC-i) valproic acid reduces the capability of M-MDSC to transdifferentiate into G-MDSC in cancer microenvironment and induces their differentiation into macrophages or DCs, thus demonstrating a novel opportunity for the selective targeting of these immunosuppressive cells (128). Along the same line, different authors (129, 130) reported on the capability of all-*trans* retinoic acid (ATRA), a natural metabolite of vitamin A, to promote MDSCs differentiation into mature myeloid cells with a mechanism dependent on ERK activation and ROS reduction. This effect was also reported in cancer patients, where ATRA administration in combination with a DC based improved the immune response to vaccination by reducing the level of MDSCs in peripheral blood (131).

Blocking the cytokines involved in the immunosuppressive microenvironment may contribute to the impairment of MDSCs activity and led to benefit for cancer patients. Treatment with the IL-1β inhibitors anakinra induced serum IL-6 decrease together with a substantial increase of PFS in high-risk smoldering or indolent MM patients (132).

Myeloid-derived suppressor cells are associated with high levels of circulating VEGF (133), however, treatment with anti-VEGF drugs such as bevacizumab did not demonstrate to affect MDSC levels in peripheral blood while increased the levels of mature DCs (134, 135). Furthermore, it is conceivable that the presence of MDSCs may represent a resistance factors to the activity of this class of drugs (30, 136, 137).

As previously described, MDSC survival and differentiation is strictly dependent on STAT3 activation, thus targeting the IL-6/Jak/Stat pathway could represent an effective strategy to block them (2). Furthermore, this pathway has a clear relevance in MM pathobiology (111, 138) and the possibility to target at the same time both MM cells and their associated immunosuppressive cells is very attractive. Different agents, including monoclonal antibodies against IL-6 or IL-6 receptor, Jak inhibitors, and STAT3 inhibitors are presently under investigation (139), and other innovative approaches, such as miRNA therapy (88), are coming out from pre-clinical research.

A different approach aims to stimulate MDSC differentiation into mature cells and to limit their expansion. As shown before G/M/GM-CSF are among the most important soluble factors involved in both processes. Different authors reported a decrease of MDSCs *in vivo* after inhibition of these molecules, associated with a relevant tumor shrinkage (140–142). However, G- and GM-CSF are widely used in different anti-cancer treatments and in MM patients: G-CSF is used to mobilize hematopoietic stem cells, while GM-CSF is often used to improve the efficacy of cancer vaccines (143, 144). Indeed, some authors reported (17) a significant increase in MDSC number in peripheral blood stem cells after G-CSF administration and hypothesized a detrimental effect on patients undergoing transplant. GM-CSF, instead, seems to be active both as an immune-adjuvant and as a MDSC inducer in a dose-dependent manner (145, 146). In our hands, however, GM-CSF resulted to be mandatory for an efficient anti-tumor response both *in vitro* and cancer patients (31, 147, 148), while the patient prognosis seemed to be related to systemic inflammation at baseline. Indeed, we hypothesized that GM-CSF administration could be detrimental in patients with a high neutrophil or monocyte count (and indirectly to MDSCs) at baseline, an event that could be related to the presence of a tumor (or its associated microenvironment) able to produce this kind of cytokines by itself and from which it depends for survival and progression. Further studies are still awaited to better clarify this apparent dualistic role of hematopoietic growth factors.

As a further attempt to reduce the immunosuppressive function of MDSCs different authors tried to down-regulate the expression of COX-2, ARG1, and iNOS and to reduce ROS formation. Celecoxib, a COX-2 selective non-steroidal anti-inflammatory drug (NSAID), has been reported to improve the efficacy of a DC-based immunotherapy and to reduce the tumor infiltration by MDSCs (82).

After demonstrating the capability of the phosphodiesterase-5 (PDE-5) tadalafil, to down-regulate *in vitro* and *in vivo* MDSC-dependent iNOS and Arg-1 (36), the same authors recently reported a case-report of a lenalidomide-resistant MM patients who achieved a reduction in MDSCs, and a 18-month lasting response with evident clinical benefits (149).

Bisphosphonates, such as zoledronic acid (ZOL), have been demonstrated to exert several effects and still represent interesting drugs in terms of anti-tumor and immune-stimulatory activity (150–157). Indeed, these agents revealed to impairs MDSCs at different levels and some authors (20) observed a decrease in the expansion of MM-induced MDSCs and a reduced capability to form osteoclasts after zoledronate treatment in mice. The mechanism responsible for this activity is thought to be dependent on the capability of zoledronate to decrease the activity of MMP9, thus reducing the bioavailability of VEGF and impairing the c-kit intracellular signaling (158–160).

A further strategy to improve cancer-associated immunosuppression consists in the selective depletion of MDSCs. Beyond the already known immunomodulatory capability of chemotherapeutic agents capecitabine and gemcitabine (124), and the recently discovered potential of doxorubicin, a drug that still play an important role in MM treatment, in selectively reducing MDSCs (161), some authors developed an RNA aptamer (162) able to block both murine or human IL-4 receptor α (IL-4Rα), critical for MDSC suppression function. The binding of the aptamer to its specific receptor led to MDSC depletion and tumor arrest of growth *in vivo*. Furthermore, others (163) recently developed an innovative method to selectively target MDSCs. By using a competitive peptide phage platform they identified peptides enriched in both M- and G-MDSCs. Subsequently, they fused the sequence of the selected peptides with the Fc portion of a murine IgG2b antibody to generate a peptibody. When used *in vivo*, these molecules, completely depleted circulating, intra-tumoral and intra-splenic MDSCs and induced a better tumor response compared to anti-Gr1 antibody. The main targets identified with this approach were found to be proteins bearing to the S100 family.

Finally, due to their major role in cancer-associated microenvironment (164, 165), miRNAs may represent a new frontier in the field of immunotherapeutic drugs (166). Unlike monoclonal antibodies or small inhibitor molecules already available, miRNAs have the great advantage to target pathways and network at multiple steps thus representing a powerful tool to target tumor-induced immune-dysfunction. As shown before, different miRNAs have already been reported to be involved in MDSC generation and function and other are currently under investigation (167). Several studies are presently ongoing to better define the future role of both miRNA replacement and inhibition in cancer therapy and immunotherapy making them a promise and a challenge for novel translational treatment strategies.

## Concluding Remarks

During the last decade, a growing effort has been devoted to understanding the role of MM-driven immunosuppression in reducing or preventing the efficacy of immunotherapy. Among others, MDSCs revealed to play a critical role in the generation of such immune dysfunctional microenvironment in different animal models and cancer patients. On these bases it is possible to speculate that the identification of molecular pathways involved in MDSC function will lead to the development of new tailored agents able to disrupt the tumor–host immunosuppressive interactions, thus improving the efficacy of both humoral and cellular immunotherapy. We now predict a new era for immunotherapy in MM, which will provide breakthrough improvements in the treatment of this important still incurable disease.

## Conflict of Interest Statement

The authors declare that the research was conducted in the absence of any commercial or financial relationships that could be construed as a potential conflict of interest.
